# Supplementary material for: Somatic increase of CCT8 mimics proteostasis of human pluripotent stem cells and extends C. elegans lifespan
Source: Nat Commun. 2016 Nov 28;7:13649. doi: 10.1038/ncomms13649 (PMC5133698; doi:10.1038/ncomms13649)
Supplement: Supplementary Information — Supplementary Figures 1-19, Supplementary Tables 1-5. [file ncomms13649-s1.pdf]

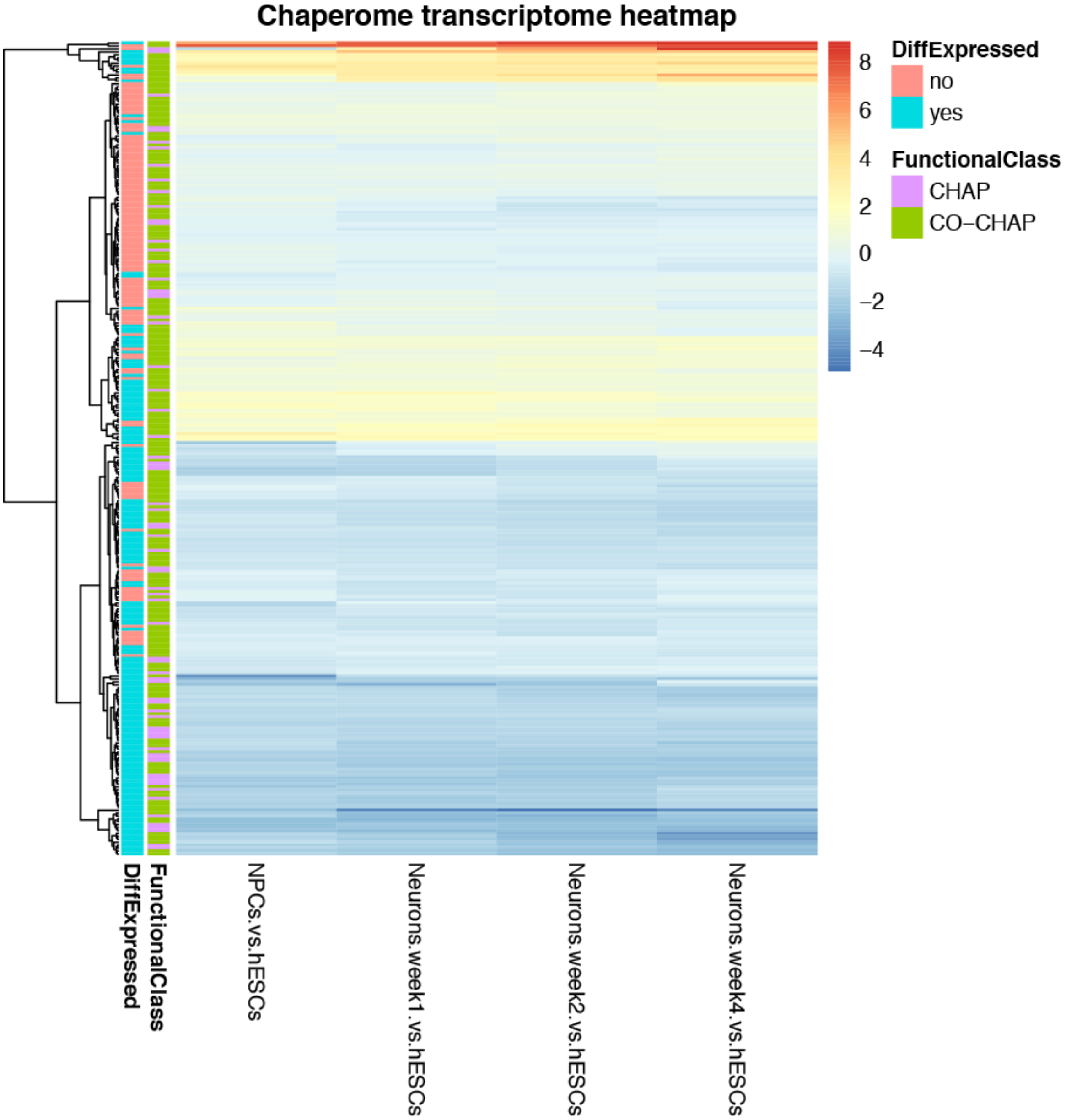

Supplementary Figure 1. Chaperome gene expression in NPCs and neurons compared to H9 hESCs. Relative gene expression abundance changes are shown on a $\log_2$ scale as heatmap. The human chaperome subtypes (chaperones (CHAP) (in purple) and co-chaperones (CO-CHAP) (in green)) are annotated as side bar. Significant abundance changes are highlighted in the respective sidebar in blue (differentially abundant) and red (not significant), respectively. Experimental group sizes were H9 hESCs (n= 3), NPCs (n= 3) and neurons at different weeks of differentiation (1 week, (n= 2), 2 weeks (n= 3) and 4 weeks (n= 2)).

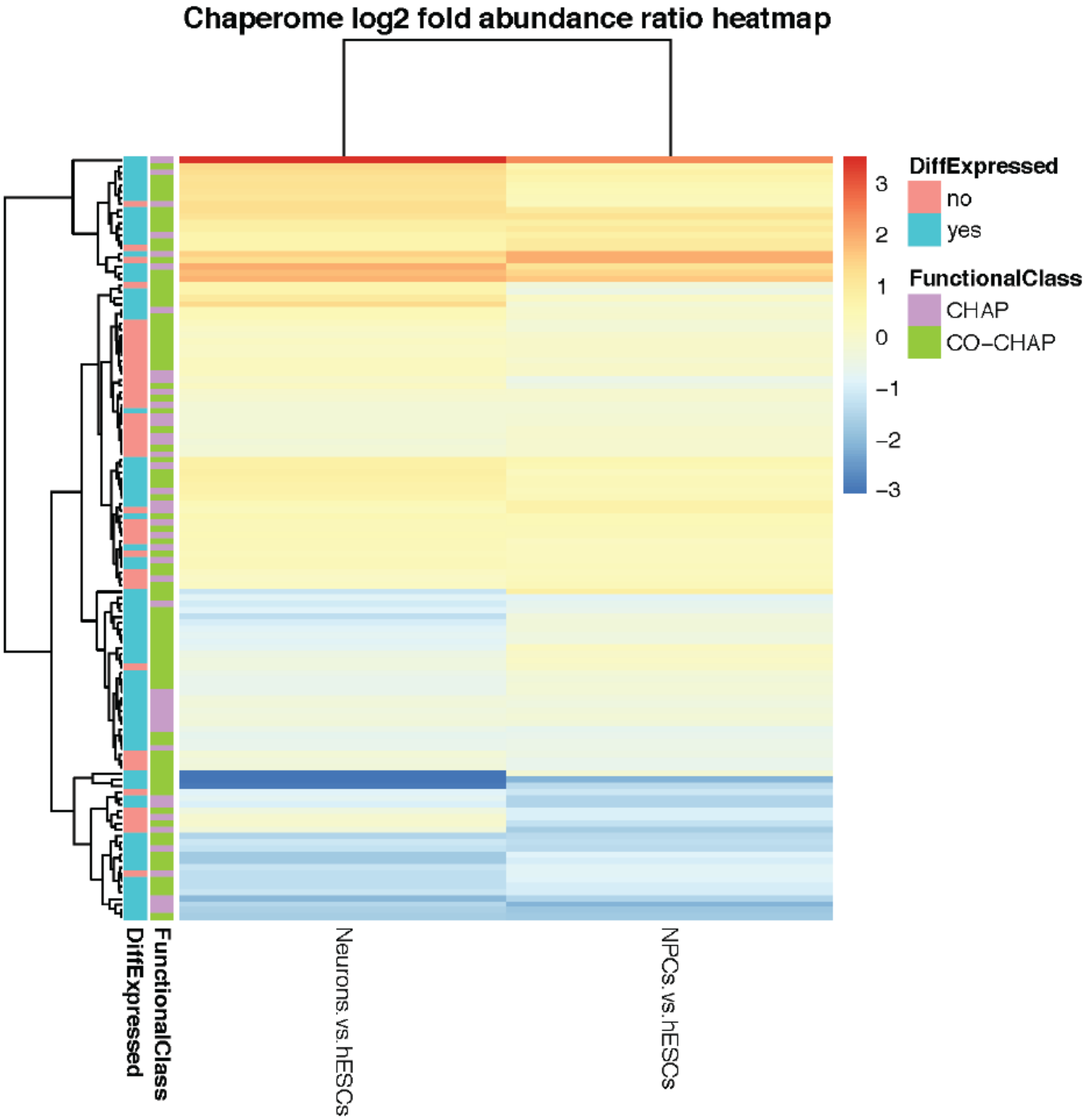

Supplementary Figure 2. Differential chaperome protein levels in NPCs and neurons compared to H9 hESCs. Relative protein abundance changes are shown on $\log_2$ scale as heatmap (hESCs (n= 9), NPCs (n= 6), neurons (4 weeks) (n= 5)). The human chaperome subtypes (chaperones (CHAP) (in purple) and co-chaperones (CO-CHAP) (in green)) are annotated as side bar. Significant abundance changes are highlighted in the respective sidebar in blue (differentially abundant) and red (not significant), respectively. We identified differentially abundant protein groups by linear modeling including cell type and experimental batch as variable using limma's moderated t-statistics framework. Adjusted P-value (q-value) of less than 0.05 is considered significant.

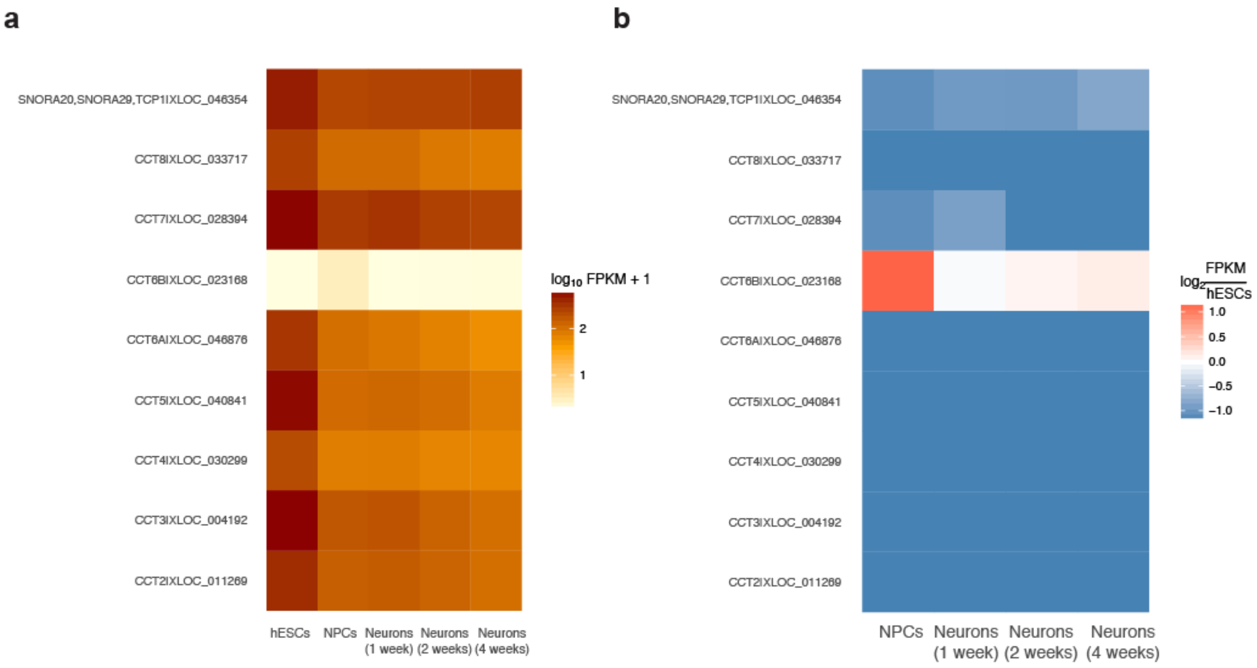

Supplementary Figure 3. Expression abundance and expression changes of CCT subunits at the mRNA level. (**a**) Absolute gene expression estimates as expressed by $\log_{10}$ FPKM+1 values. (**b**) CCT relative expression to hESCs as expressed by $\log_2$ FPKM Condition – $\log_2$ FPKM hESCs.

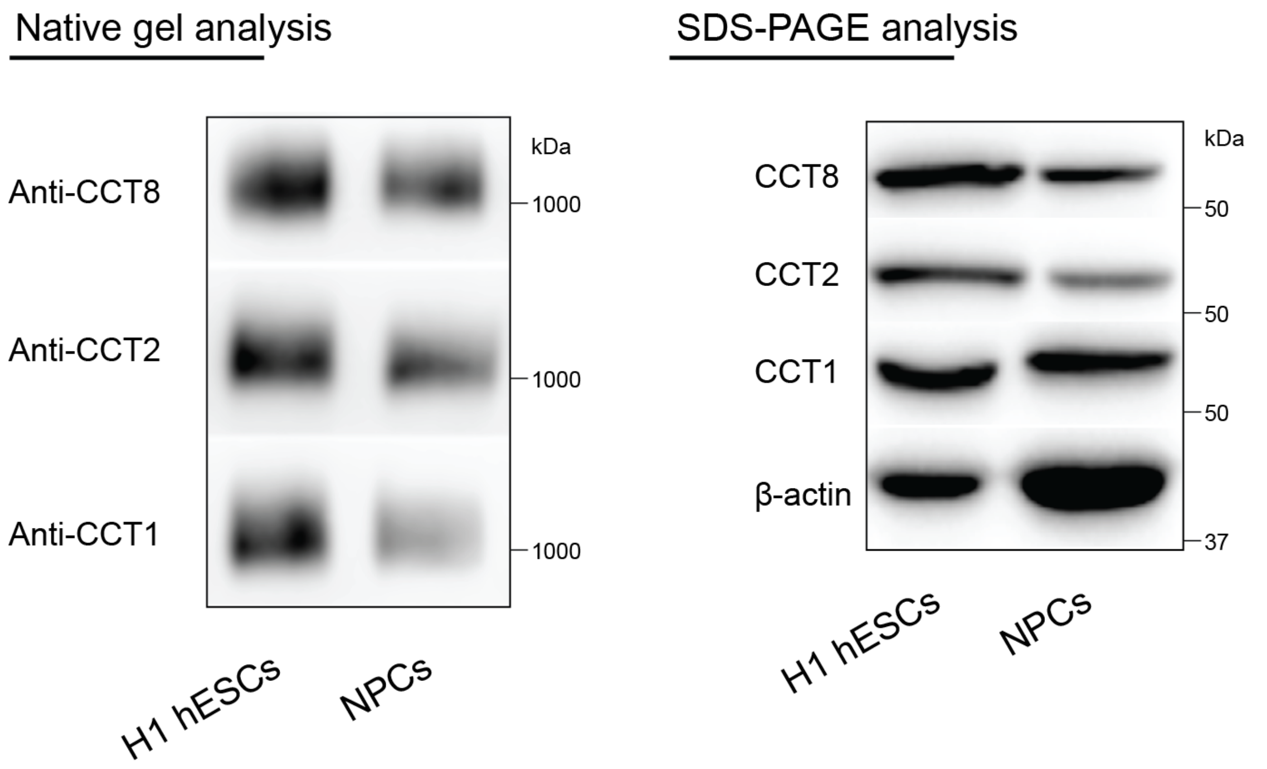

Supplementary Figure 4. H1 hESCs have enhanced assembly of TRiC/CCT complexes. Native gel electrophoresis of H1 hESCs extracts followed by immunoblotting with CCT antibodies. In the right panel, extracts were resolved by SDS-PAGE and immunoblotting for analysis of total CCT subunit levels and β-actin loading control. The images are representative of two independent experiments.

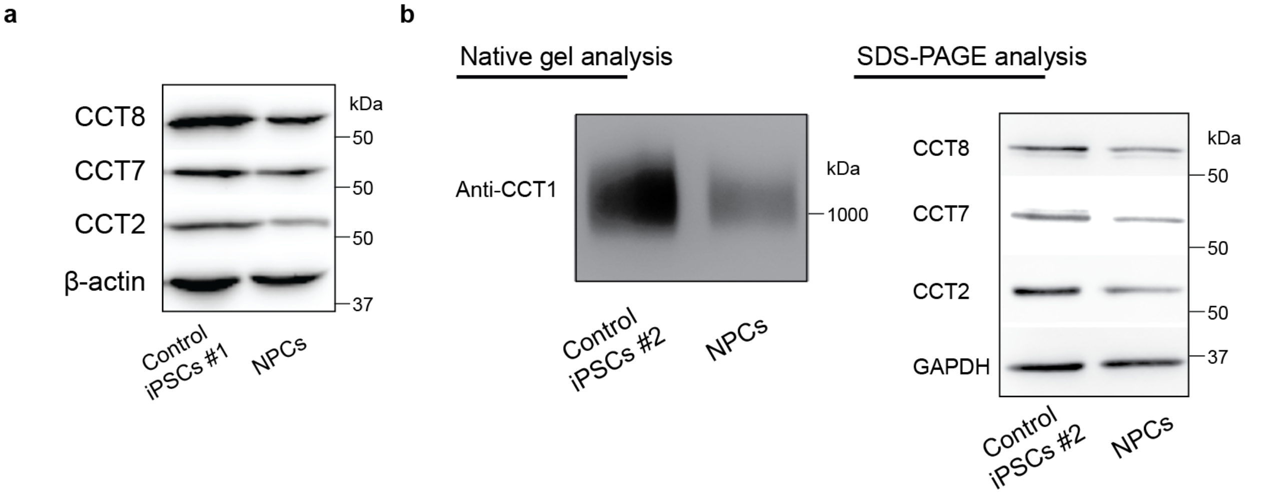

Supplementary Figure 5. iPSCs exhibit increased expression of CCT subunits and TRiC/CCT assembly. (**a**) Western blot analysis of control iPSCs #1 and their NPC counterparts with antibodies to CCT8, CCT7 and CCT2. β-actin is the loading control. The images are representative of two independent experiments. (**b**) Native gel electrophoresis of control iPSCs (line #2) and their NPC counterparts followed by immunoblotting with CCT1 antibody. In the right panel, extracts were resolved by SDS-PAGE and immunoblotting for analysis of total CCT subunit levels and GAPDH loading control. The images are representative of three independent experiments.

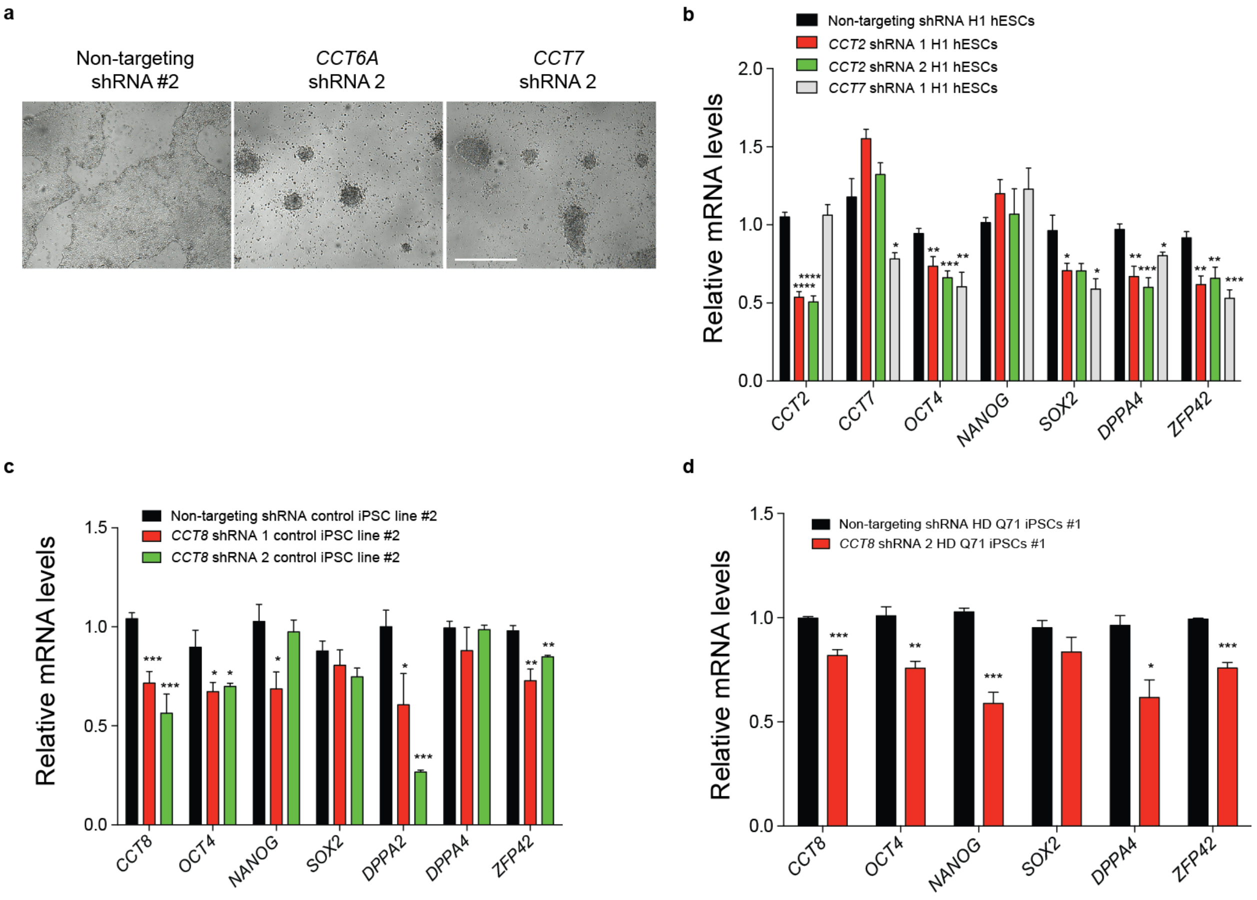

Supplementary Figure 6. Mild knockdown of *CCT* subunits affects the expression of pluripotency markers in independent pluripotent stem cell lines. (**a**) Representative brightfield images of two independent experiments. Acute knockdown of CCT subunits induces cell death and detachment of H9 hESCs. Scale bar represents 500 µm. (**b**) Real Time PCR analysis of pluripotency markers in H1 hESCs upon mild knockdown of CCT subunits. Graph (relative expression to NT shRNA) represents the mean ± s.e.m. (n= 3 independent experiments). (**c**) Real Time PCR analysis of pluripotency markers in control iPSC line #2 upon mild knockdown of CCT8. Graph (relative expression to NT shRNA) represents the mean ± s.e.m. (n= 3 independent experiments). (**d**) Real Time PCR analysis in HD Q71 iPSC line #1 upon mild knockdown of CCT8. Graph (relative expression to NT shRNA) represents the mean ± s.e.m. of four independent experiments. All the statistical comparisons were made by Student's t-test for unpaired samples. P-value: *(P<0.05), **(P<0.01), *** (P<0.001), **** (P<0.0001).

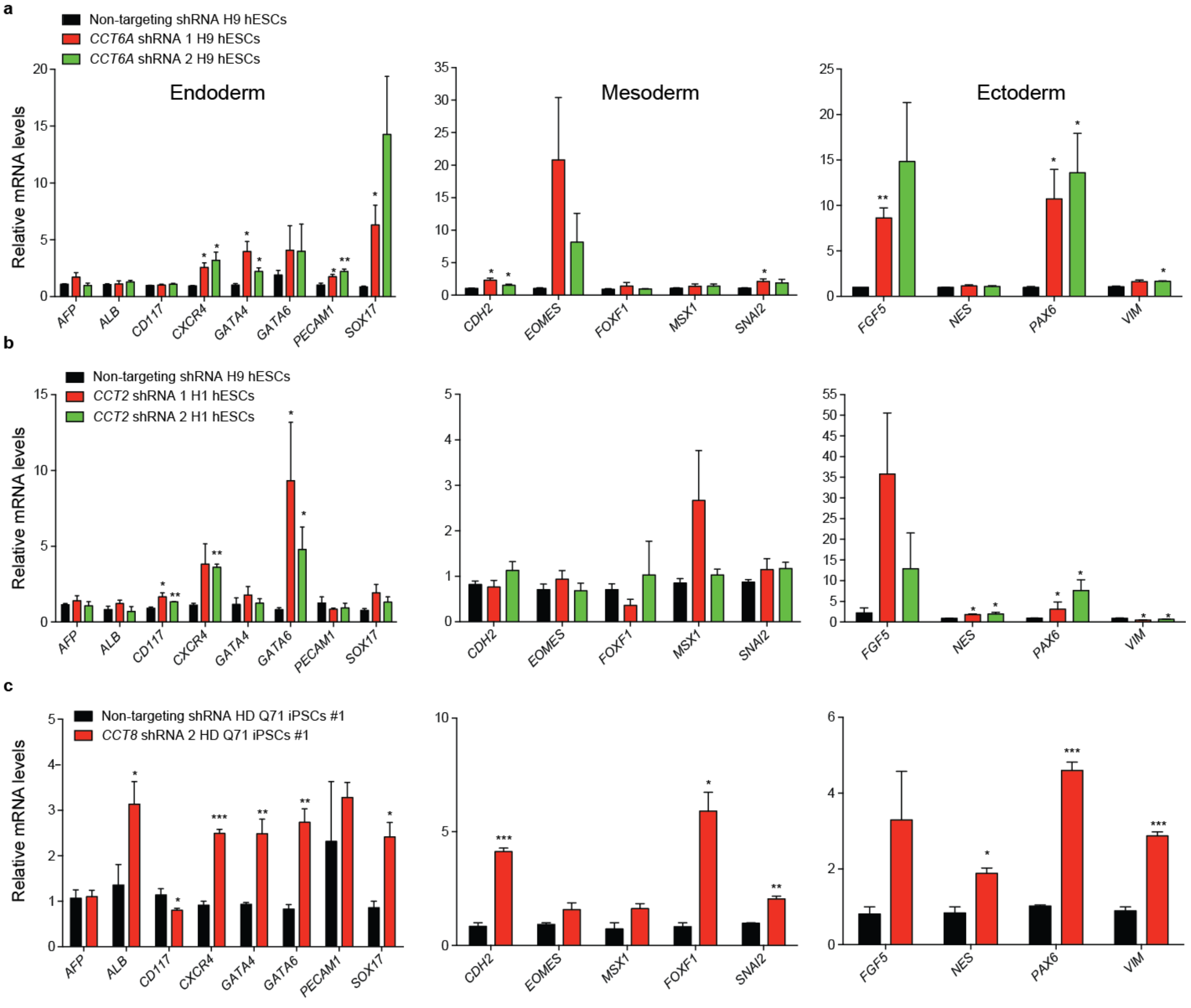

Supplementary Figure 7. Knockdown of *CCT* subunits affects the expression of germ layer markers in different cell lines. (**a**) Real Time PCR analysis of germ layer markers in H9 hESCs (relative expression to NT shRNA). Graph represents the mean ± s.e.m. of four independent experiments. (**b**) Real Time PCR analysis of germ layer markers in H1 hESCs (relative expression to NT shRNA). Graph represents the mean ± s.e.m. of three independent experiments. (**c**) Real Time PCR analysis of germ layer markers in HD Q71 iPSC line #1 (relative expression to NT shRNA). Graph represents the mean ± s.e.m. of four independent experiments. All the statistical comparisons were made by Student's t-test for unpaired samples. P-value: *(P<0.05), **(P<0.01), *** (P<0.001).

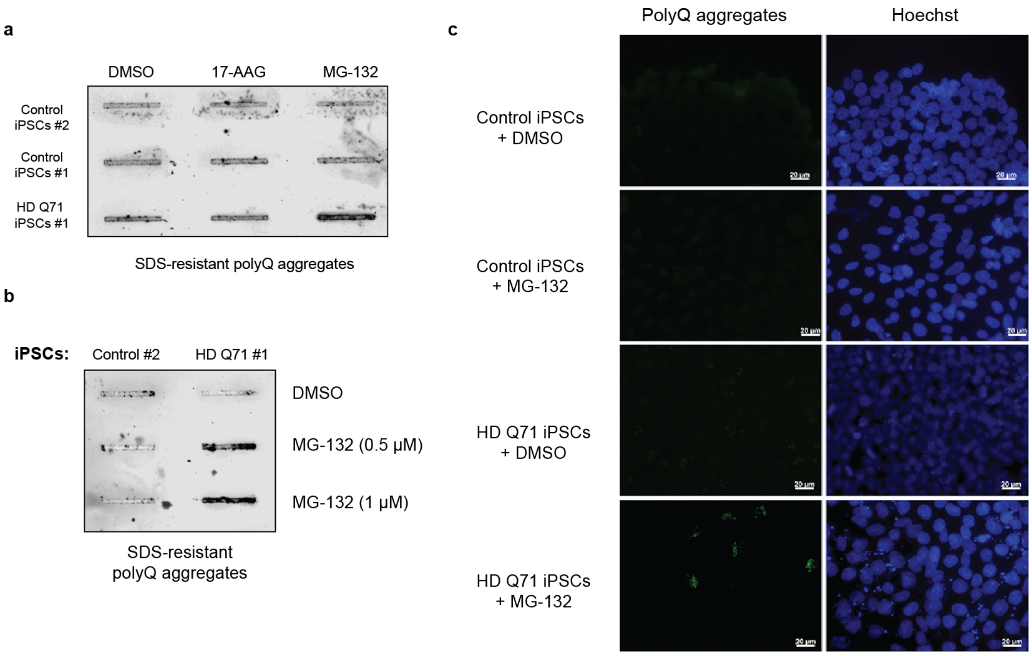

Supplementary Figure 8. Proteasome inhibition triggers the accumulation of polyQ aggregates in HD-iPSCs. (**a**) Filter trap analysis shows that the treatment of HD-iPSCs with 17-N-Allylamino-17-demethoxygeldanamycin (17-AAG), a HSP90 inhibitor, does not affect polyQ aggregation (detected by anti-polyQ-expansion diseases marker antibody). Cells were treated either with 250 nM 17-AAG for 48 h or 5 µM MG-132 (proteasome inhibitor) for 16 h. The images are representative of two independent experiments. (**b**) Filter trap experiments show that proteasome inhibition with MG-132 for 12 h results in increased levels of polyQ aggregates in the pellet of HD Q71-iPSCs (line #1). However, proteasome inhibition does not induce accumulation of polyQ aggregates in control iPSC line #2 (polyQ21). The images are representative of three independent experiments. (**c**) Immunocytochemistry of control iPSC line #2 (Q21) and HD Q71-iPSC line #1 treated with 5 µM MG-132 for 12 h. Expanded polyQ antibody was used to detect polyQ aggregates. Cell nuclei were stained with Hoechst 33342. Scale bar represents 20 µm. The images are representative of three independent experiments.

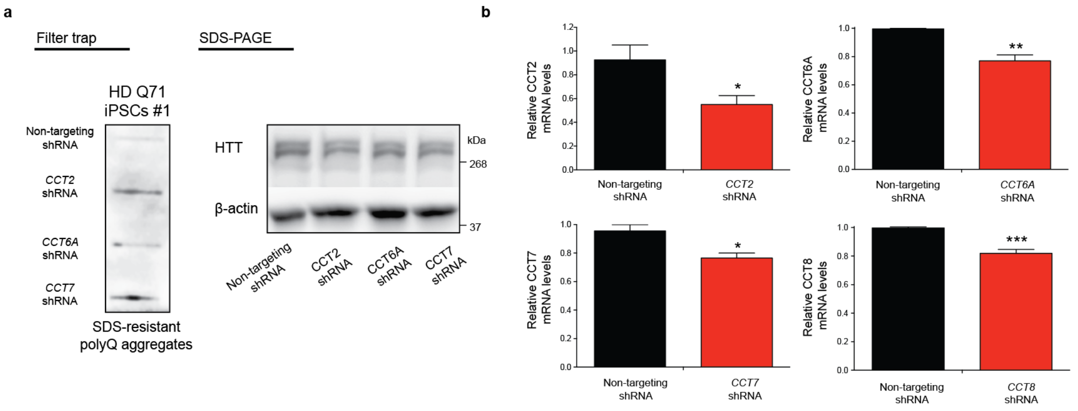

Supplementary Figure 9. Knockdown of different CCT subunits triggers the accumulation of polyQ aggregates. (**a**) Filter trap analysis of HD polyQ71 iPSC line #1 upon knockdown of different CCT subunits. Right panel: SDS-PAGE analysis with antibodies to HTT and β-actin loading control. The images are representative of three independent experiments. (**b**) CCT knockdown levels in HD polyQ71 iPSC line #1 (corresponding to filter trap experiments shown in **Figure 4d** and **Supplementary Figure 9a**). Graphs (relative expression to non-targeting shRNA) represent the mean ± s.e.m of 4 independent experiments. All the statistical comparisons were made by Student's t-test for unpaired samples. P-value: *(P<0.05), **(P<0.01), ***(P<0.001).

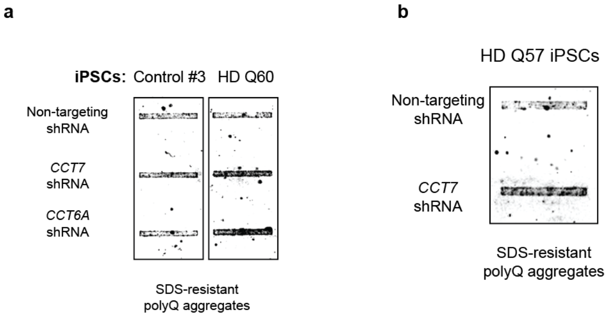

Supplementary Figure 10. Knockdown of CCT subunits triggers the accumulation of polyQ aggregates in different HD iPSC lines. Filter trap analysis shows that loss of CCT subunits results in increased polyQ aggregates in both (**a**) HD polyQ60 iPSCs and (**b**) HD polyQ57 iPSCs. In contrast, loss of CCT subunits did not change the levels of polyQ aggregates in control iPSC line #3 (polyQ33). The images are representative of two biological replicates of a single experiment.

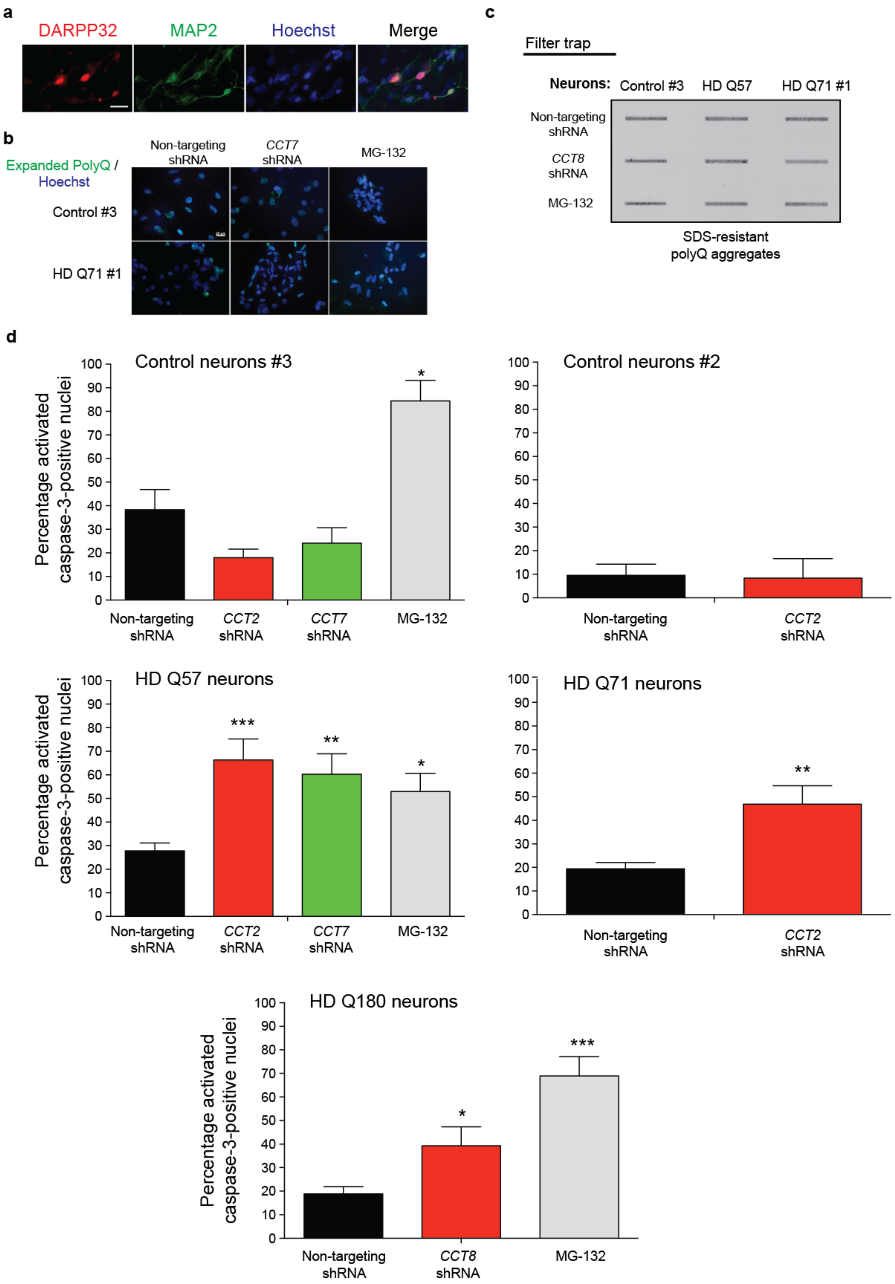

Supplementary Figure 11. Dysfunction of TRiC/CCT induces activation of caspase-3 in neurons derived from HD-iPSCs. (**a**) Immunocytochemistry of HD Q57 neurons. DARPP32 staining was used as a marker of striatal neurons. MAP2 and Hoechst 33342 staining were used as markers of neurons and nuclei, respectively. Scale bar represents 50 µm. Among MAP2-positive neurons, approximately 50% were also DARPP32-positive. The images are representative of two independent experiments. We obtained similar efficiency of differentiation with other lines (*i.e.*, Control #2, Control #3, HD Q71 line #2 and HD Q180). (**b**) Immunocytochemistry of neurons derived from iPSCs with expanded polyQ antibody and Hoechst 33342. Scale bar represents 20 µm. The images are representative of three independent experiments. (**c**) Filter trap analysis of neurons derived from iPSCs. Expanded polyQ antibody was used to detect polyQ aggregates. MG-132: 5 µM MG-132 for 24 h. The images are representative of two biological replicates. (**d**) Percentage of activated caspase-3-positive nuclei. Graph represents the mean ± s.e.m. of the percentage observed in 4 independent neuronal differentiation experiments (we assessed approximately 150 total nuclei in each independent experiment). MG-132: 5 µM MG-132 for 24 h. Activated caspase-3-positive nuclei were stained with antibody to cleaved caspase-3. Total number of cells was evidenced after staining of nuclei with Hoechst 33342. All the statistical comparisons were made by Student's t-test for unpaired samples. P-value: *(P<0.05), **(P<0.01), *** (P<0.001).

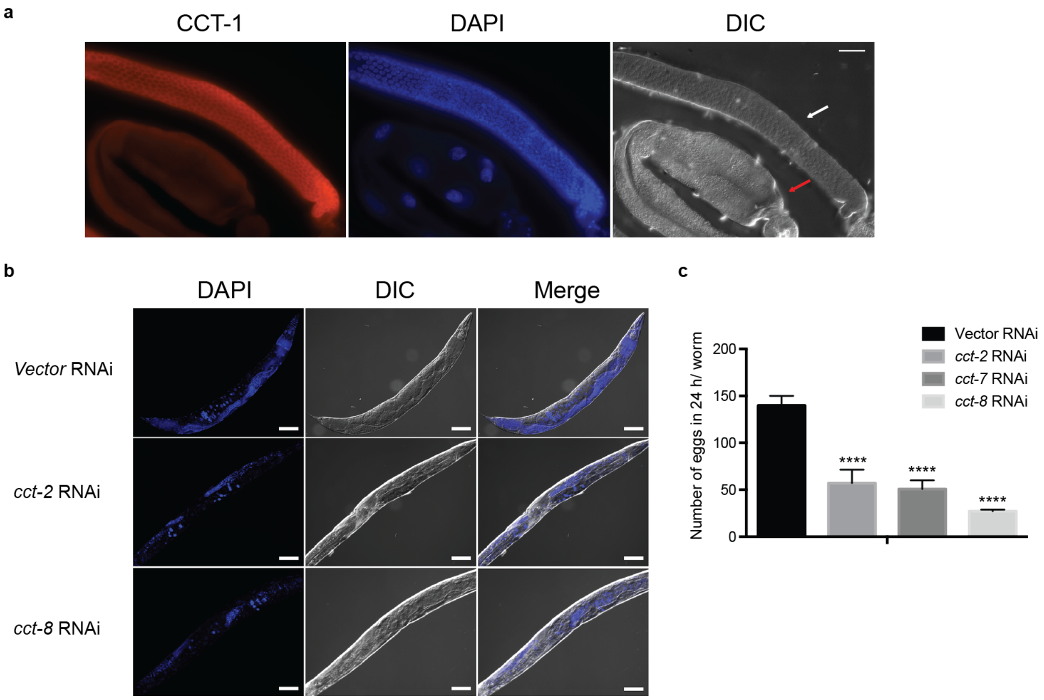

Supplementary Figure 12. Knockdown of CCT subunits destabilizes *C. elegans* germline. (**a**) Gonad and intestine immunostaining with CCT-1 antibody from day 1-adult wild-type worms. Cell nuclei were stained with DAPI. White arrow indicates gonad and red arrow indicates intestine. Scale bar represents 20 µm. DIC= differential interference contrast. The images are representative of two independent experiments. (**b**) Nuclei staining with DAPI of day 4 adult wild-type worms. Knockdown of *cct* subunits during adulthood reduces the number of germ line cells. RNAi was initiated at day 1 of adulthood. Scale bar represents 100 µm. The images are representative of two independent experiments. (**c**) Number of eggs laid per worm during 24 h. RNAi was initiated at day 1 of adulthood. We started counting the number of laid eggs after 1 day of RNAi treatment. Graph represents the mean ± s.e.m. of 5 independent experiments (in each independent experiment, we counted and averaged the number of eggs laid by 6 worms for each treatment). All the statistical comparisons were made by Student's t-test for unpaired samples. P-value: **** ($P<0.0001$).

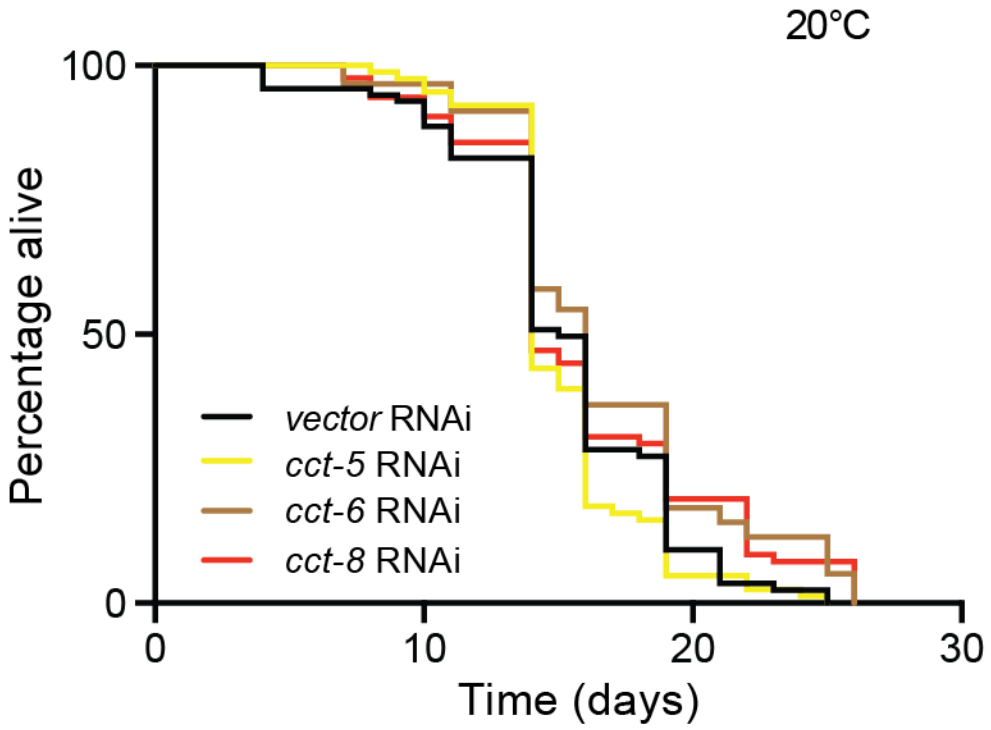

Supplementary Figure 13. Loss of *cct* subunits during adulthood does not significantly decrease lifespan of wild-type worms at 20°C. *vector* RNAi: median= 15, n= 83/96; *cct-5* RNAi: median= 14, n= 78/96; *cct-6* RNAi: median= 16, n= 77/96; *cct-8* RNAi: median= 14, n= 81/96. All the statistical comparisons were made using the log-rank (Mantel–Cox) method.

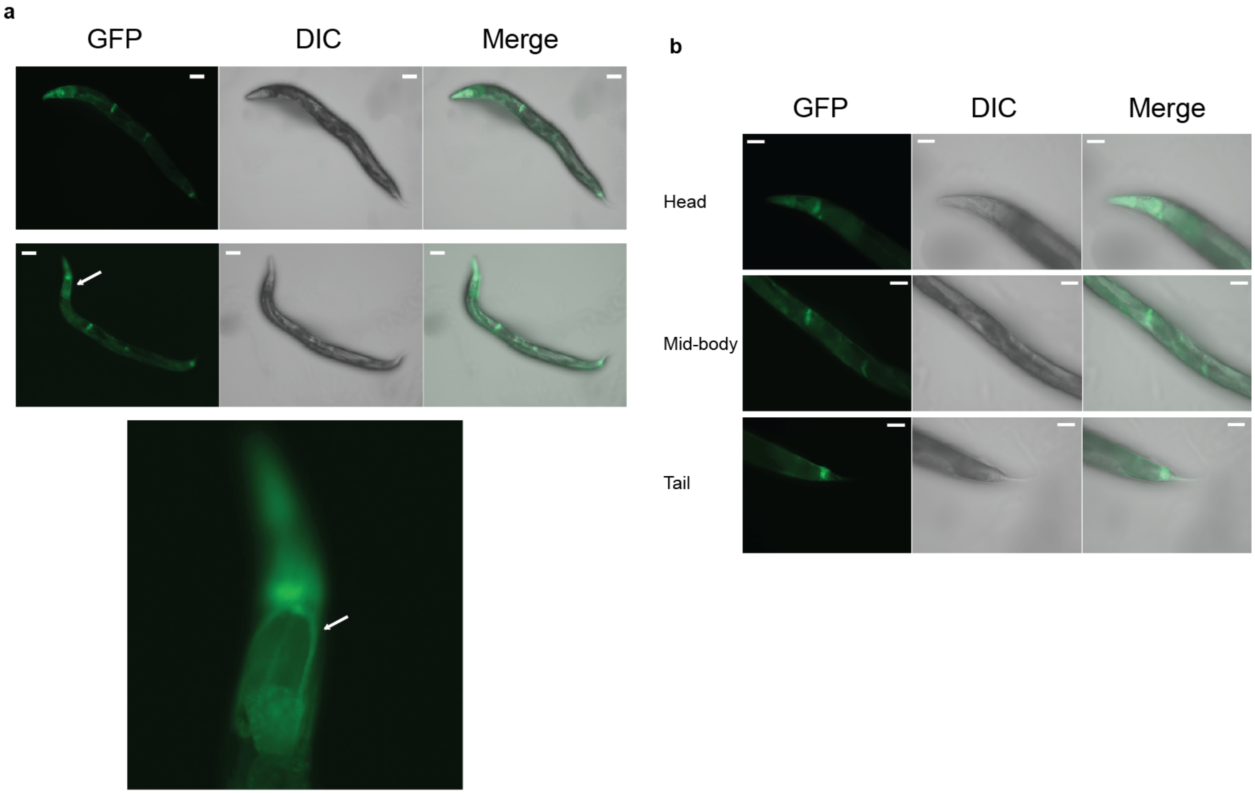

Supplementary Figure 14. *cct-8* is widely expressed in somatic tissues. (**a**) Representative images of GFP expressed under control of the *cct-8* promoter in whole adult worm. Scale bar represents 100 µm. Arrow indicates neurons. (**b**) Adult expression of GFP under *cct-8* promoter in the head, mid-body and tail regions. Scale bar = 50 µm. The images are representative of three independent experiments.

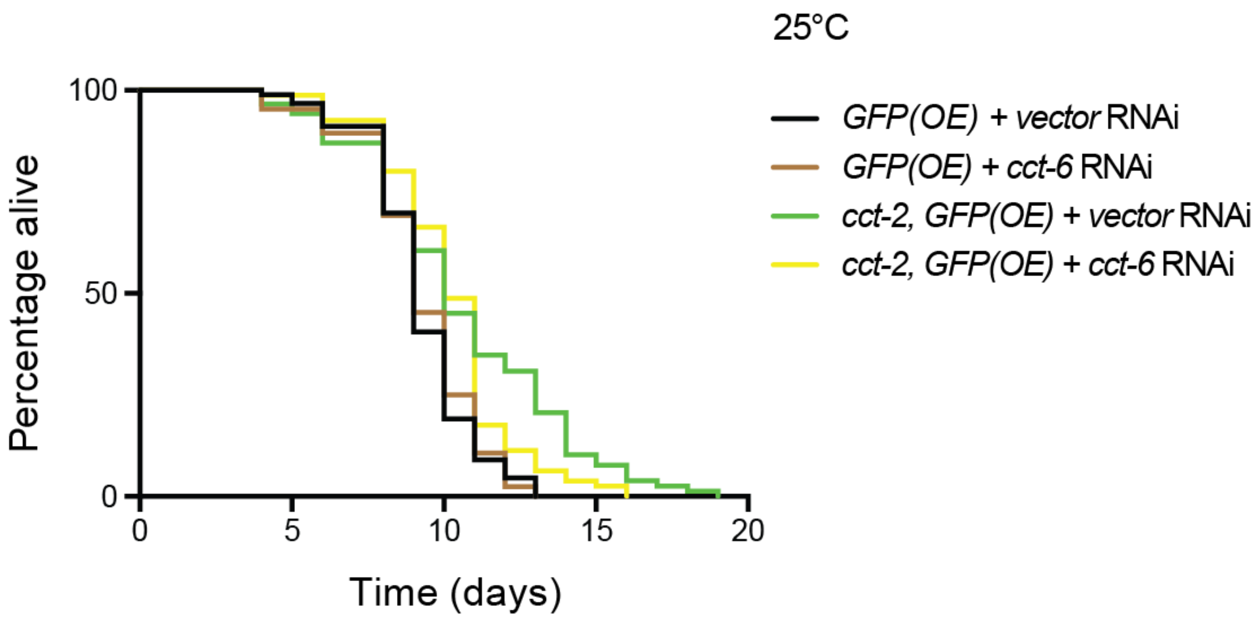

Supplementary Figure 15. Knockdown of *cct-6* reduces the lifespan extension induced by *cct-2(OE)* at 25°C. RNAi was initiated at day 1 of adulthood. *GFP(OE)* fed *empty vector* RNAi bacteria (median= 9, n= 89/96) *versus GFP(OE)* fed *cct-6* RNAi bacteria (median= 9, n= 84/96): log rank, P= 0.7502. *cct-2, GFP(OE)* fed *empty vector* RNAi bacteria (median= 11, n= 89/96) *versus cct-2, GFP(OE)* fed *cct-6* RNAi bacteria (median= 10, n= 84/96): log rank, P= 0.0335. All the statistical comparisons were made using the log-rank (Mantel–Cox) method.

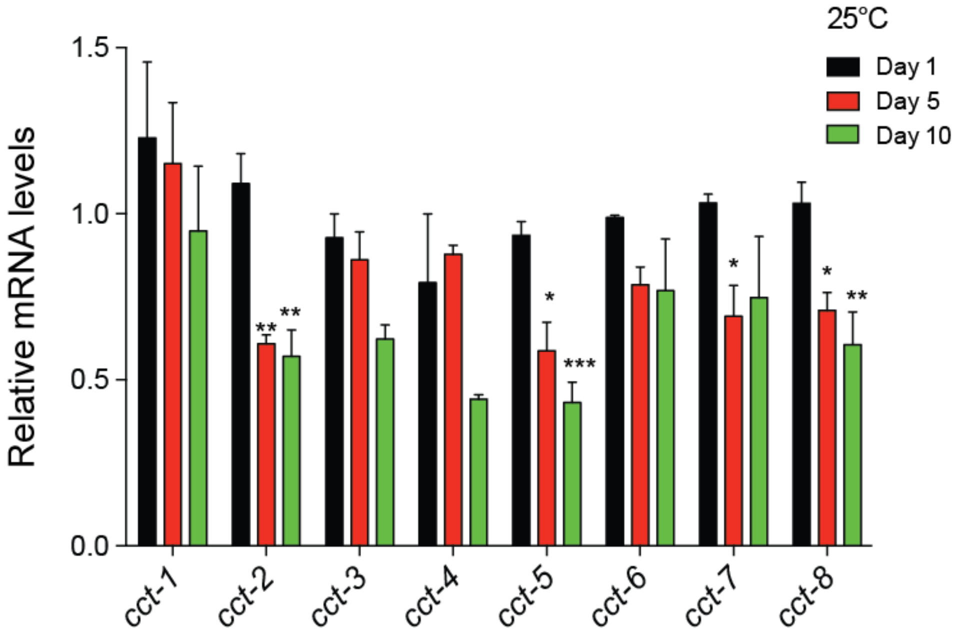

Supplementary Figure 16. The expression of specific *cct* subunits decreases with age at 25°C. Data represent the mean ± s.e.m. of the relative expression levels to day 1 adult worms grown at 25°C (n= 4). These experiments were performed with the sterile control strain *fer-15(b26)II;fem-1(hc17).* Statistical comparisons were made by Student's t-test for unpaired samples. P-value: *(P<0.05), **(P<0.01), *** (P<0.001).

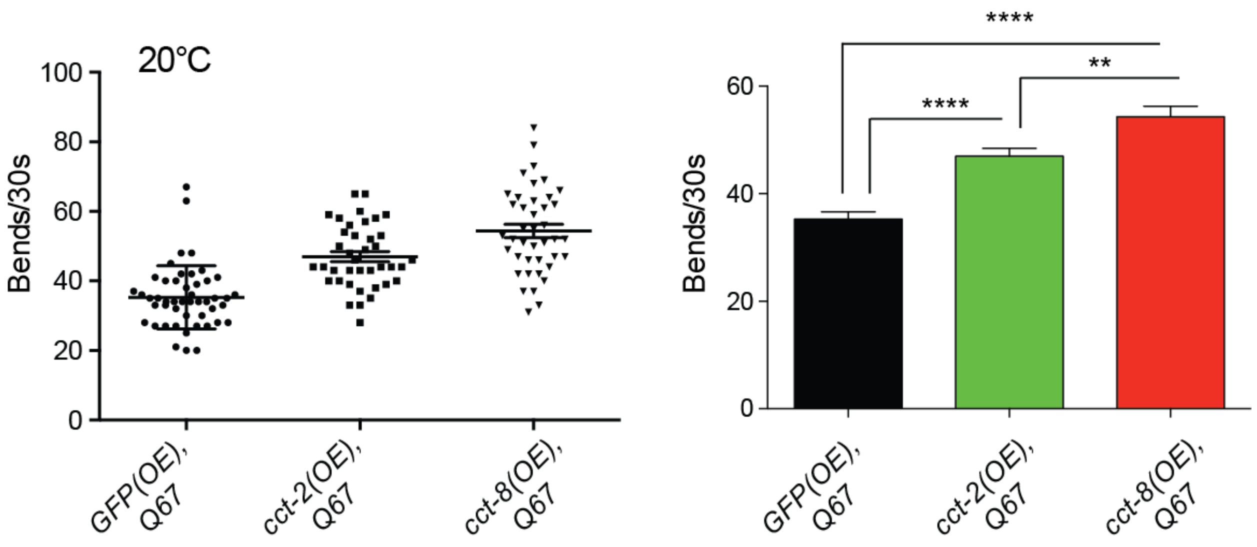

Supplementary Figure 17. Ectopic expression of *cct-8* and *cct-2* improves motility in polyQ67 worms at 20°C. In the left panel, each point represents the average thrashing rate of a single 3 day-adult animal over a period of 30 s. In the right panel, bar graphs represent average ± s.e.m. of these data (*GFP(OE);Q67* (n= 48), *cct-2,GFP(OE);Q67* (n= 39), *cct-8,GFP(OE);Q67* (n= 41)). All the statistical comparisons were made by Student's t-test for unpaired samples. P-value: **(P<0.01), **** (P<0.0001).

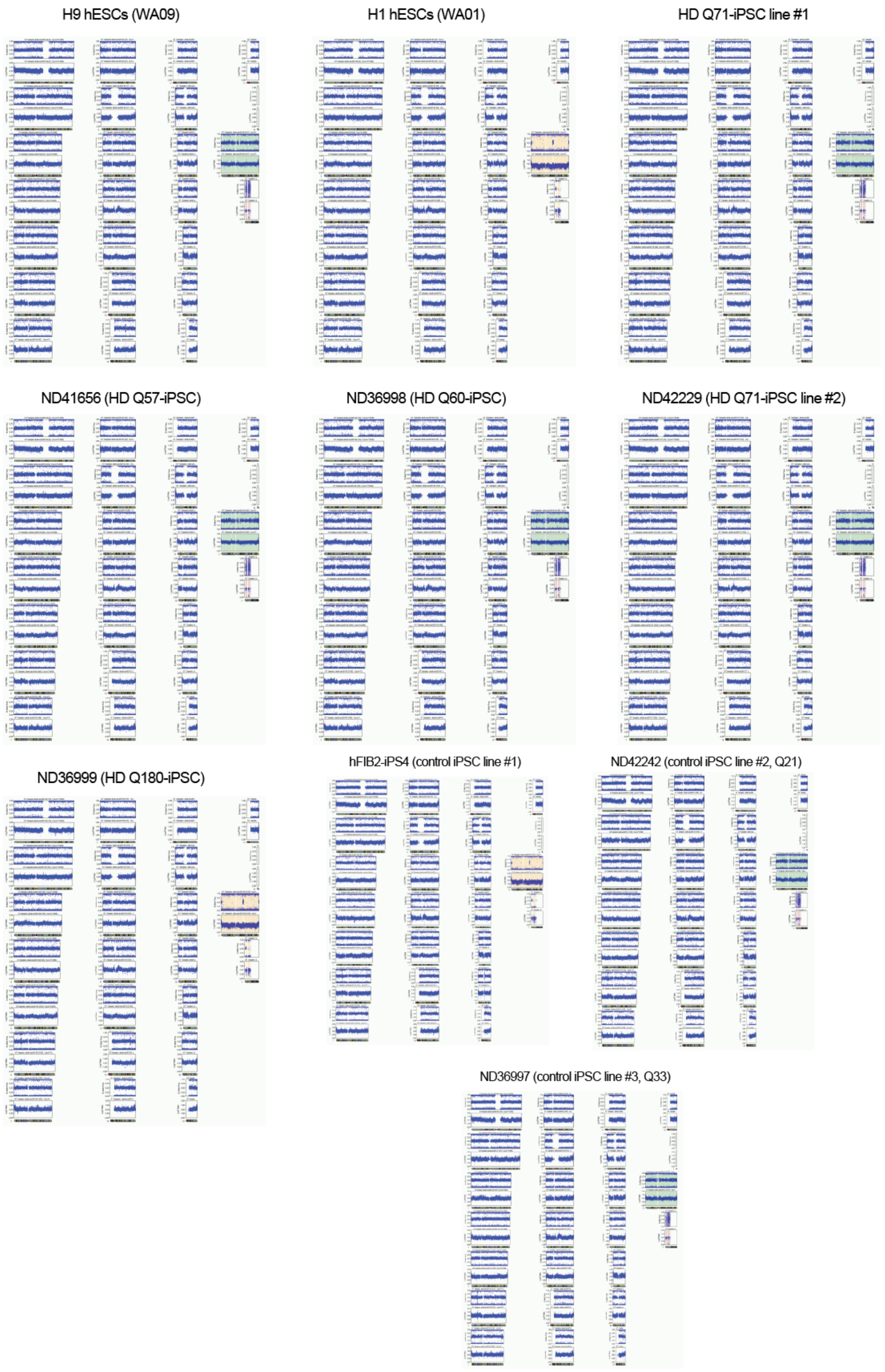

Supplementary Figure 18. SNP genotyping of hESCs and iPSCs used in this study.

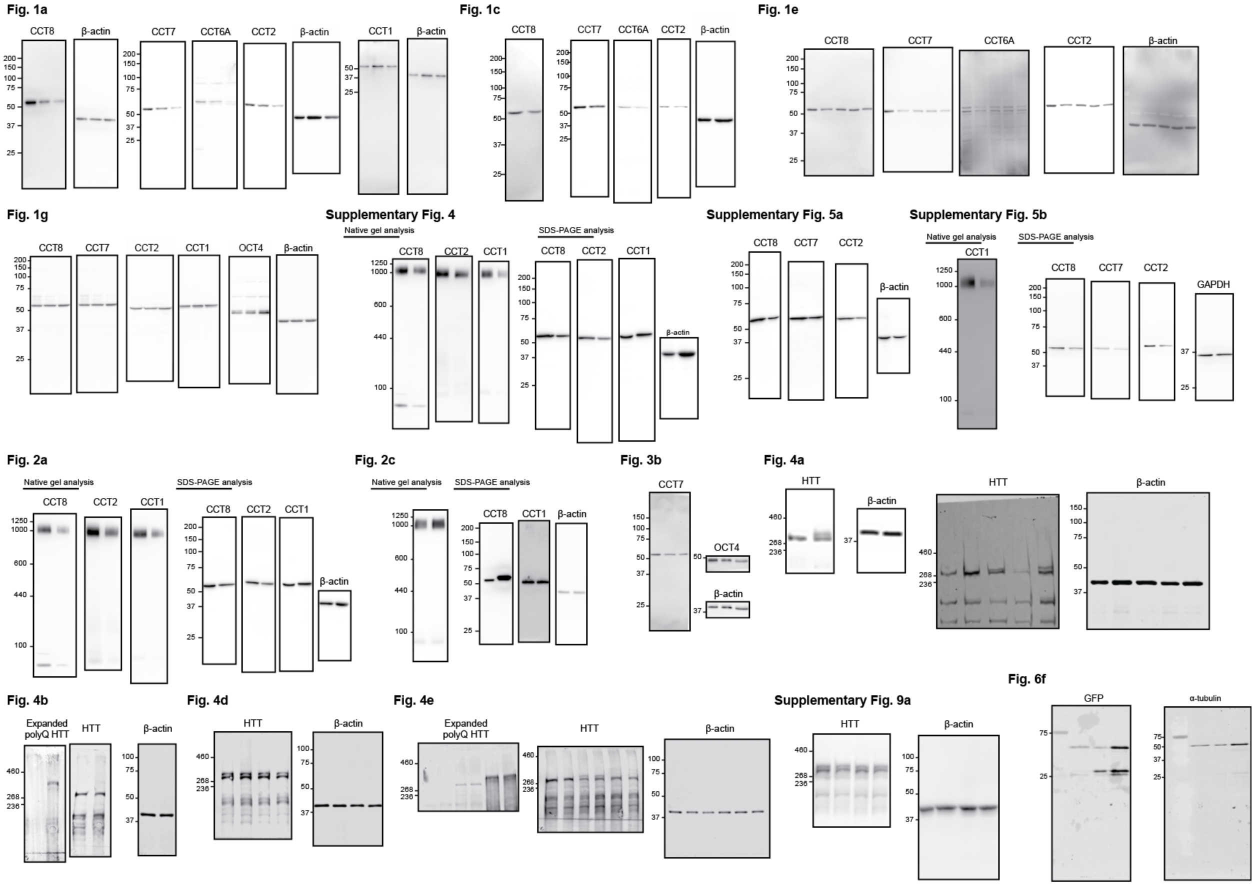

Supplementary Figure 19. Uncropped images of most important western blots. Uncropped images are presented with molecular weight ladders.

Gene names	NPCs		Neurons	
	T-test difference	q-value	T-test difference	q-value
**TCP1**	-0.04	0.731476	-0.22	0.055274
**CCT2**	-0.18	0.143481	-0.23	0.061077
**CCT3**	-0.30	0.028618	-0.43	0.002554
**CCT4**	-0.37	0.011644	-0.37	0.013717
**CCT5**	-0.19	0.1434813	-0.17	0.091828
**CCT6A**	-0.24	0.077786	-0.29	0.035591
**CCT7**	-0.09	0.516294	0.02	0.900321
**CCT8**	-0.37	0.002746	-0.30	0.013732

Supplementary Table 1. Quantitative proteomic analysis of CCT subunit levels comparing H9 hESCs with their NPC and neuronal counterparts. hESCs exhibit increased levels of specific CCT subunits. We used limma's moderated t-test to contrast NPCs (n= 6) and neurons (n= 5) versus hESCs (n= 9). Relative abundance differences are calculated from the $\log_2$ of label-free quantification (LFQ) values (LFQ NPCs/hESCs and LFQ Neurons/hESCs).

Locus ID	Alleles	
	H9 hESCs	H1 hESCs
D5S818	11, 12	9, 11
D13S317	9, 9	8, 11
D7S820	9, 11	8, 12
D16S539	12, 13	9,13
vWA	17, 17	15, 17
TH01	9.3, 9.3	9.3, 9.3
TP0X	10, 11	8,11
CSF1P0	11, 11	12, 13

Supplementary Table 2. STR analysis of H9 and H1 hESCs.

	Allele size in base pairs							
	**TPOX**	**CSFIPO**	**D5S818**	**THO1**	**vWA**	**D10S526**	**D17S1303**	**D16S539**
	Allele1, Allele2	Allele1, Allele2	Allele1, Allele2	Allele1, Allele2	Allele1, Allele2	Allele1, Allele2	Allele1, Allele2	Allele1, Allele2
**GMO3621**	228,232	310, 314	147,151	159,171	145,167	249,253	235,239	154,161
**ND36998**	228,232	310, 314	147,151	159,171	145,167	249,253	235,239	154,161
**GMO9197**	232,237	306,310	147,151	155,171	135,145	239	231,235	154, 159
**ND36999**	232, 237	306,310	147,151	155,171	135,145	239	231,235	154, 159
**GMO2183**	228,232	310, 315	147,151	168,171	145,159	237,241	231,235	150, 154
**ND36997**	228,232	310,315	147,151	168,171	145,159	237,241	231,235	150, 154
**GMO4281**	232,245	306,310	147,151	164,168	145,154	237,241	219,223	154,159
**HD519**	232,245	306,310	147,151	164,168	145,154	237,241	219,223	154,159
**ND42229**	232,245	306,310	147,151	164,168	145,154	237,241	219,223	154,159
**ND30014**	241,245	305,318	150,152	168,172	138,141	237,241	223,227	154, 158
**ND42242**	241,245	305,318	150,152	168,172	139,141	237,241	223,227	154, 158
**ND33392**	237,241	309,314	142,147	164,171	140,145	229,232	223,235	154, 159
**ND41656**	237,240	309,314	142,147	164,171	140,145	229,232	223,235	154, 159

Supplementary Table 3. Confirmation of genetic identity of iPSC lines with the corresponding parental fibroblasts by STR analysis.

List of primers used for qPCR assays- Human cells

Housekeeping genes

Gene	Forward (5' → 3')	Reverse (5' → 3')
ACTB	CTGGCACCCAGCACAATG	CCGATCCACACGGAGTACTTG
GAPDH	GCACCGTCAAGGCTGAGAAC	GGATCTCGCTCCTGGAAGATG

Pluripotency markers

Gene	Forward (5' → 3')	Reverse (5' → 3')
0CT4	GGAGGAAGCTGACAACAATGAAA	GGCCTGCACGAGGGTTT
NANOG	AAATCTAAGAGGTGGCAGAAAAACA	GCCTTCTGCGTCACACCATT
SOX2	TGCGAGCGCTGCACAT	TCATGAGCGTCTTGGTTTTCC
DPPA4	CTGGTGCCAACAATTGAAGCT	AGGCACACAGGCGCTTATATG
DPPA2	GTACTAATGGCAAGAAAATCGAAGTTT	GCCGTTGTTCAGGGTAAGCA
ZFP42	CCTGCAGGCGGAAATAGAAC	GCACACATAGCCATCACATAAGG

Markers for the germ layers

Gene	Forward (5' → 3')	Reverse (5' → 3')
AFP	GAGGGAGCGGCTGACATTATT	ACCAGGGTTTACTGGAGTCATTTC
ALB	TGAGGTTGCTCATCGGTTTAAA	GCAATCAACACCAAGGCTTTG
CD117	CCAAGGCCGACAAAAGGA	GGCGGGAGTCACATCTCTTTC
CXCR4	GGCCGACCTCCTCTTTGTC	TTGCCACGGCATCAACTG
GATA4	TCCGTGTCCCAGACGTTCTC	GAGAGGACAGGGTGGATGGA
GATA6	AGCGCGTGCCTTCATCA	GTGGTAGTTGTGGTGTGACAGTTG
PECAM	GGAGTCCAGCCGCATATCC	GCTTGGAAAATAGTTCTGTTATGTTGA
SOX17	TGGCGCAGCAGAATCCA	CGACTTGCCCAGCATCTTG
FGF5	ACGAGGAGTTTTCAGCAACAAAT	TTGGCACTTGCATGGAGTTTT
NES	TGAAGGGCAATCACAACAGG	TGACCCCAACATGACCTCTG
PAX6	CATACCAAGCGTGTCATCAATAAAC	TGCGCCCATCTGTTGCT
VIM	TCTGCCTCTTCCAAACTTTTCC	AACCAGAGGGAGTGAATCCAGAT
CDH2	CAGCAACGACGGGTTAGTCA	TGCAGCAACAGTAAGGACAAACA
EOMES	ATGCAGGGCAACAAAATGTATG	GTCTCATCCAGTGGGAACCAGTA
FOXF1	AGCCGTATCTGCACCAGAACA	ACTCCTTTCGGTCACACATGCT
MSX1	CTCCGCAAACACAAGACGAAC	CACATGGGCCGTGTAGAGTC
GATA6	AGCGCGTGCCTTCATCA	GTGGTAGTTGTGGTGTGACAGTTG
SNAI2	TGCGGCAAGGCGTTTT	CTCCCCCGTGTGAGTTCTAATG

CCT subunits

Gene	Forward (5' → 3')	Reverse (5' → 3')
CCT2	AAGCCACGAAGGCTGCAA	TCATCGGAACCATGATCAACTG
CCT5	CGGATAAGTGCCCCACCTTA	TCCAGTGCGTCGGCAAA
CCT6A	TGGCCAGAACATCTCTTCGTACT	AGTCCACTACAGCCTCTGTTAAGACA
CCT7	GTGGCATGGACAAGCTTATTGTAG	CAGAATTGTGGCCCCATCA
CCT8	ACCCGGAGGTGGAGCAA	GGACATGTCTCTCCATATGATGTGA

Supplementary Table 4. List of primers used for qPCR assays with human cell extracts.

List of primers used for qPCR assays-*C. elegans*

**Housekeeping genes**	**Forward (5' → 3')**	**Reverse (5' → 3')**
cdc-42	CTGCTGGACAGGAAGATTACG	CTCGGACATTCTCGAATGAAG
pmp-3	GTTCCCGTGTTCATCACTCAT	ACACCGTCGAGAAGCTGTAGA

**Chaperones**	**Forward (5' → 3')**	**Reverse (5' → 3')**
cct-1	CCAGCCAAGCTTGAGGCTAT	TCAATGCGGCGTTTGGTA
cct-2	GGACTTGACTCGGCTGAACTTG	CGATGTCGATTCCCATATTGTG
cct-3	GCGTGAGAGTGGCCATCAG	CGGCGATCGTCTTGCAA
cct-4	TGCACTTGAGCTCATTCCATACA	AACAGTGTGAATTGGTGACAATCC
cct-5	TCTTGGAAGCAAAATCGTAAACC	ACAGCATCGACAGCGATTTCT
cct-6	ACGAAATGGCGATTCAACATC	GTCGTCTTGTGCCGTCGAA
cct-7	TTGAGGGCAAGGATCAAGCT	CGTGGGATGATCTCGAAAGC
cct-8	CGGCCGCTTTGTTGGA	TGTGGTGTCATTCCCATATGGA
enpl-1	GAAACAAGAGAGGAAGATTCGATCA	GCTTTTGAGCGGAGCTCCTT

Supplementary Table 5. List of primers used for qPCR assays with *C. elegans* extracts.
